# Antioxidant and Immune Stimulating Effects of *Allium cepa* Skin in the RAW 264.7 Cells and in the C57BL/6 Mouse Immunosuppressed by Cyclophosphamide

**DOI:** 10.3390/antiox12040892

**Published:** 2023-04-06

**Authors:** Ji-Su Kim, Eun-Byeol Lee, Ji-Hye Choi, Jieun Jung, Un-Yul Jeong, Ui-Jin Bae, Hwan-Hee Jang, Shin-Young Park, Youn-Soo Cha, Sung-Hyen Lee

**Affiliations:** 1 Functional Food Division, Department of Agro-Food Resources, National Institute of Agricultural Sciences, Rural Development Administration, Wanju 55365, Republic of Korea; ver0218@korea.kr (J.-S.K.); dmsqufdl1029@naver.com (E.-B.L.); jyyye@jbnu.ac.kr (J.-H.C.); jjempm@korea.kr (J.J.); juw325@naver.com (U.-Y.J.); euijin0230@korea.kr (U.-J.B.); rapture19@korea.kr (H.-H.J.); 2 Fermented and Processed Food Science Division, Department of Agro-Food Resources, National Institute of Agricultural Sciences, Rural Development Administration, Wanju 55365, Republic of Korea; soyoenj@korea.kr; 3 Department of Food Science and Human Nutrition, Jeonbuk National University, 567 Baekje-Daero, Jeonju 54896, Republic of Korea; cha8@jbnu.ac.kr

**Keywords:** *Allium cepa* skin, antioxidant, immune-stimulating, in vitro, in vivo

## Abstract

*Allium cepa* L. (onion) has been reported to have various pharmacological effects, such as preventing heart disease, and improving antimicrobial activity and immunological effects. The Republic of Korea produced 1,195,563 tons of onions (2022). The flesh of onion is used as food while the onion skin (OS) is thrown away as an agro-food by-product and is considered to induce environmental pollution. Thus, we hypothesize that increasing usage of OS as functional food material could help protect from the environment pollution. The antioxidant effects and immune-enhancing effects of OS were evaluated as functional activities of OS. In this study, OS showed high 1,1-diphenyl-2-picrylhydrazyl (DPPH) and 2,2-azino-bis(3-ethylbenzothiazoline-6-sulfonic acid) (ABTS) radical scavenging activities and xanthine oxidase (XO) inhibitory activity. The antioxidant activities increased in a dose-dependent manner. The IC_50_ values of DPPH, ABTS radical scavenging activity, and XO inhibitory activity were 954.9 μg/mL, 28.0 μg/mL, and 10.7 μg/mL, respectively. Superoxide dismutase and catalase activities of OS in RAW 264.7 cells were higher than those of the media control. There was no cytotoxicity of OS found in RAW 264.7 cells. Nitric oxide and cytokines (IL-1β, IL-6, IFN-γ, and TNF-α) concentrations in RAW 264.7 cells significantly increased in a dose dependent manner. Immune-stimulating effects of OS were evaluated in immunosuppressed mice induced by cyclophosphamide. White blood cell count and the B cell proliferation of splenocytes were higher in OS100 (OS extract 100 mg/kg body weight) and OS200 (OS extract 200 mg/kg body weight) groups than in the negative control (NC) group. Serum IgG and cytokine (IL-1β and IFN-γ) levels were also higher in OS100 and OS200 groups than in the NC group. OS treatment increased NK cell activity compared with the NC group. The results suggested that OS can improve antioxidant and immune stimulating effects. The use of OS as functional supplement can reduce the agro-food by-product and it may contribute to carbon neutrality.

## 1. Introduction

Oxidative stress is caused by the imbalance between oxidants and antioxidants [1]. The typical oxidants are reactive oxygen species (ROS) and reactive nitrogen species (RNS). They induce cancer, aging, inflammation, and cardiovascular disease. Antioxidants are important to protect the body and prevent the damage of oxidative stress. Recently, compounds obtained from foods have been evaluated for antioxidant effects. Bioactive compounds, such as polyphenols, terpenoids, flavonoids, and vitamins, protect the cellular damage from oxidants [2]. Antioxidant enzymes, including superoxide dismutase (SOD), catalase (CAT), and glutathione peroxidases (GPx), have beneficial roles in chronic diseases [1].

Immunity is a biological defense mechanism that occurs to eliminate a substance threating body or destroying homeostasis. It is broadly divided into innate and adaptive immunes. Innate immune consists of cytokine and white blood cells that induce macrophage and natural killer (NK) cells, and perform primary defense before adaptive immune. Macrophages perform an important part in the innate immune system, and its role in the host’s defense mechanism is the first to recognize the invasion of external substances and cell-mediated immunity [3]. Activated macrophages cause morphological changes of cells, motility, and proliferation. Additionally, they produce NO and cytokines (interleukin-1β; IL-1β, interleukin-6; IL-6, interferon-γ; IFN-γ, and tumor necrosis factor-α; TNF-α), and these build up immune response and suppress the growth of cancer cells [3,4]. NK cells show autogenous cytolytic activity against cells under stress, such as tumor cells and virus-infected cells, and secrete various cytokines, such as IFN-γ and TNF-α [5]. Adaptive immune, called specific immunity, consists of cell-mediated immunity involving T cells and humoral immunity by antibodies produced by B cells. It starts when T cells recognize the antigen presented by predation of antigen presenting cells. Thus, the body is designed to maintain homeostasis by appropriately countering the innate or adaptive immune system against the invasion or infection of foreign substances, such as external antigens [6].

Onion (*Allium cepa* L.) has various nutrients, including protein, fiber, potassium, folate, and vitamin C. Onion has many health benefits, such as potential in preventing heart disease, decreasing of blood lipid level, and improving antioxidant and anti-atherogenic effects [7,8,9]. *Allium* sp. has antimicrobial activity and immunological effects and onion especially improves anti-cancer properties [7,8]. Flesh onion is usually considered an edible portion, but the onion skins (OS) are often discarded and may cause environmental pollution [10]. OS extract has been used to synthesize nanoparticles for biomedical applications [11] or chemical compounds, such as bisenols [12]. OS derived carbon was utilized as an electrode material for highly efficient and low-cost energy storage devices [13]. Antioxidant and anti-inflammatory effects of solvent and hot water extracts from OS have been studied, and they were reported as valuable materials for the industrial utilization [14,15]. The hot water extract has higher yield and is safer in bioassays with lower toxicity than the ethanol extract [16].

This is the first study showing the antioxidant and immune stimulating effects of hot water extract from OS in both in vitro and in vivo, which are very important factors to protect the body and keep people’s health. The antioxidant and immunomodulatory effects of OS were conducted in RAW 264.7 cells and C57BL/6 mice immunosuppressed by cyclophosphamide (CPA). In this study, OS showed high antioxidant and immunomodulatory effects. Thus, it can be used for functional food, which improves antioxidant and immunomodulatory activities, and may prevent the earth from the environmental pollution through upcycling by-products and carbon neutrality.

## 2. Materials and Methods

### 2.1. Preparation of Plant Material and Reagents

#### 2.1.1. Sample Preparation for the Experiment

OS was purchased from Muan farm (Jeonnam province, Republic of Korea), and its extract was supplied by Jeonbuk Institute for Food-Bioindustry (Jeonju, Republic of Korea). Briefly, OS was extracted twice with 20-fold volume of hot water at 90 °C for 8 h, filtered through 25 µm (Suin Co. Gwangju, Republic of Korea), and concentrated at 65 °C and 600–700 mH using the extracting and concentration system (HS Tech., Seongnam, Republic of Korea). The OS extract was then frozen and lyophilized (PVTFD 300R, Ilsin Lab, Yangju, Republic of Korea), and the extraction yield of OS was 11.5%. The specimen (RDA-21-ACS-01) was stored at −80 °C in the Department of Agricultural Food Resources, National Academy of Agricultural Science, Rural Development Administration.

#### 2.1.2. Reagents for the Experiment

Quercetin, Folin–Ciocalteu’s phenol reagent, gallic acid, sodium carbonate (Na_2_CO_3_), 1,1-diphenyl-2-picrylhydrazyl (DPPH), 2,2’-azino-bis(3-ethylbenzothiazoline-6-sulfonic acid) (ABTS), potassium persulfate, potassium phosphate buffer (pH 7.5), xanthine, xanthine oxidase, lipopolysaccharide (LPS), cyclophosphamide (CPA), β-glucan, histopaque, and concanavalin A (Con A) were purchased from Sigma-Aldrich Co. (St. Louis, MO, USA). RAW 264.7 cells were purchased from the Korean Cell Line Bank (Seoul, Republic of Korea). Dulbecco’s modified Eagle medium (DMEM), fetal bovine serum (FBS), penicillin-streptomycin, and Hank’s balanced salt solution (HBSS) were purchased from Gibco (New York, NY, USA). MTS solution and Griess reagent system were purchased from Promega Co. (Fitchburg, WI, USA). SOD colorimetric activity kit and catalase colorimetric activity kit were purchased from Invitrogen Co. (Carlsbad, CA, USA). ELISA kits (IL-1β (ab197742), IFN-γ (ab282874), TNF-α (ab285327), and IgG (ab157719)) were purchased from abcam (London, UK). PURY RNA Plus kit and amfiRivert cDNA Synthesis Platinum Master Mix were purchased from GenDEPOT (Katy, TX, USA). Primers (GAPDH (QT01658692), IL-1β (QT01048355), IL-6 (QT00098875), IFN-γ (QT01038821), and TNF-α (QT00104006)) for cytokine expression were purchased from QIAGEN GmbH (Hilden, Germany). Twenty-five micrometer filter paper and 0.45 μm membrane filter were purchased from Suin Co. (Gwangu, Republic of Korea). Tube with K_2_EDTA was purchased from BD (Franklin Lakes, NJ, USA). Murine NK cell Activity ELISA test kit was purchased from NKMAX Co., Ltd. (Seongnam, Republic of Korea). Forty micrometer nylon cell strainer was purchased from BD Biosciences (San Jose, CA, USA).

### 2.2. Evaluation of Functional Compounds and Antioxidant Activity

#### 2.2.1. Quercetin Concentration

Quercetin concentration was measured by modified method of Lee et al. [17]. OS (5.18 mg) was sonicated in 50 mL of solvent (methanol:formic acid:water = 50:5:45) at 65 °C for 60 min. The sample was left at room temperature (RT) for cooling and filtered with 0.45 μm membrane filter and used as a test solution. Standard solutions of quercetin were prepared and analyzed to obtain a standard calibration curve for calculating the quercetin content in OS. For analysis conditions, an Agilent 1260 Infinity Binary LC (Agilent Technology, Santa Clara, CA, USA) was used for the HPLC system. The column is a Zorbax Eclipse Plus C18 UG 120 (4.6 × 250 mm, 5 μm). Mobile phase A was 5% formic acid and mobile phase B was MeOH for a gradient elution of mobile phase A and mobile B. The UV length was 360 nm, the injection volume was 10 μL, the column temperature was 40 °C, and the flow rate was 0.8 mL/min (Table 1). 

#### 2.2.2. Total Phenolic Content

Total phenolic content (TPC) assay was conducted by the method of Jeong et al. [18]. Twenty microliters sample was mixed with 80 μL of DW and 40 μL of 100% Folin–Ciocalteu’s phenol reagent in a 96 well plate, and it was incubated at RT for 3 min. The solution was mixed with 60 μL of 10% Na_2_CO_3_ and reacted for 2 h. The absorbance was measured at 725 nm using a microplate reader (Molecular Devices, San Jose, CA, USA). Methanol and gallic acid were used as sample blank and standard, respectively. TPC was expressed as mg gallic acid equivalent (GAE)/g of sample.

#### 2.2.3. DPPH Radical Scavenging Activity 

1,1-diphenyl-2-picrylhydrazyl (DPPH) radical scavenging activity was measured by the method of Brand-Williams [19]. Fifty microliters sample was mixed with 200 μL of DPPH solution, then the mixture was incubated at RT for 30 min in the dark condition. Absorbance was measured at 517 nm using a microplate reader (Molecular Devices). DPPH radical scavenging activity was expressed as % and a 50% inhibitory concentration (IC_50_) value. DPPH radical scavenging ability was calculated by the following formula:

$$\mathrm{DPPH}\;\mathrm{radical}\;\mathrm{scavenging}\;\mathrm{activity}\;\left(\%\right)=\left(1-\frac{\mathrm{Absorbance}\;\mathrm{of}\;\mathrm{sample}-\mathrm{Absorbance}\;\mathrm{of}\;\mathrm{sample}\;\mathrm{blank}}{\mathrm{Absorbance}\;\mathrm{of}\;\mathrm{control}}\right)\times 100$$

#### 2.2.4. ABTS Radical Scavenging Activity

2,2’-azino-bis(3-ethylbenzothiazoline-6-sulfonic acid) (ABTS) radical scavenging activity was measured by the method of Re et al. [20]. ABTS (7.4 mM) and potassium persulfate (2.6 mM) were mixed and kept at 4 °C for 24 h in a dark condition. The ABTS solution was diluted to get an absorbance of 0.70 ± 0.02 at 760 nm. A total of 50 μL sample was mixed with 200 μL of ABTS solution, then the mixture was incubated at RT for 10 min in the dark condition. Absorbance was measured at 517 nm using a microplate reader (Molecular Devices). Sample blank was prepared by mixing the methanol with sample and the methanol was used as a control. ABTS radical scavenging activity was expressed as % and IC_50_ value. ABTS radical scavenging ability was calculated by the following formula.

$$\mathrm{ABTS}\;\mathrm{radical}\;\mathrm{scavenging}\;\mathrm{activity}\;\left(\%\right)=\left(1-\frac{\mathrm{Absorbance}\;\mathrm{of}\;\mathrm{sample}-\mathrm{Absorbance}\;\mathrm{of}\;\mathrm{sample}\;\mathrm{blank}}{\mathrm{Absorbance}\;\mathrm{of}\;\mathrm{control}}\right)\times 100$$

#### 2.2.5. Xanthine Oxidase Inhibitory Activity 

Samples were mixed with 0.6 mL of 0.1 M potassium phosphate buffer (pH 7.5), and 0.2 mL of the substrate solution in 2 mM xanthine. The mixture was added with 0.1 mL of xanthine oxidase (0.2 U/mL) and incubated at 37 °C for 10 min, and 1 N HCl was added to stop the reaction. Absorbance was measured at 517 nm using a microplate reader. The inhibitory activity against xanthine oxidase (XO) was expressed as a percentage (%) and IC_50_ value.

### 2.3. Cell Experiment for Evaluations of Antioxidant and Immunomodulatory Effects 

#### 2.3.1. Cell Viability

The mouse macrophage strain RAW 264.7 cells were cultured in DMEM containing 10% heat-inactivation FBS and 1% penicillin-streptomycin in the incubator with 5% CO_2_ at 37 °C. RAW 264.7 cells were seeded (2 × 10^5^ cells/well) in 96 well plates and incubated at 37 °C for 4 h, and were treated with various concentration of OS and incubated for 48 h. Twenty microliters of MTS solution were added in each well and incubated for 2 h in a dark condition. The absorbance was measured at 490 nm using a microplate reader (Molecular Devices).

$$\mathrm{Cell}\;\mathrm{viability}\;\left(\%\right)=\frac{\mathrm{Absorbance}\;\mathrm{of}\;\mathrm{sample}}{\mathrm{Absorbance}\;\mathrm{of}\;\mathrm{control}}\;\times \;100$$

#### 2.3.2. Superoxide Dismutase Activity

RAW 264.7 cells were treated with OS (62.5 μg/mL, 125 μg/mL, and 250 μg/mL) and incubated for 48 h. The supernatant was collected by centrifugation (15,000 rpm, 10 min, 4 °C). SOD activity was evaluated according to the SOD colorimetric activity kit protocol (EIASODC, Invitrogen Co., Carlsbad, CA, USA). In brief, each 10 μL of supernatant was added to 50 μL of the substrate solution with 25 μL of a xanthine oxidase solution in a new plate and the plate was kept at RT for 20 min. The absorbance was measured at 450 nm using a microplate reader (Molecular Devices). The SOD activity was calculated by the standard curve.

#### 2.3.3. Catalase Activity

RAW 264.7 cells were treated with OS (62.5 μg/mL, 125 μg/mL, and 250 μg/mL) and incubated for 48 h. The supernatant was collected by centrifugation (15,000 rpm, 10 min, 4 °C). CAT activity was measured according to the catalase colorimetric activity kit manual (EIACATC, Invitrogen Co.). Briefly, 25 μL of samples were mixed with 25 μL of hydrogen peroxide reagent and were reacted at RT for 30 min. The reaction product was added to 25 μL of substrate solution and HRP solution and was incubated at 25 °C for 15 min. The absorbance was measured at 560 nm using a microplate reader (Molecular Devices). CAT activity was calculated by the standard curve.

#### 2.3.4. Nitric Oxide Concentration

RAW 264.7 cells were seeded in 96 well plates at 2 × 10^5^ cells/well and were treated with various concentrations (31.13 μg/mL, 62.25 μg/mL, 125 μg/mL, 250 μg/mL, and 500 μg/mL) of OS and LPS (1 ng/mL), respectively, which was used as a positive control. NO concentration of sample was measured using the Griess reagent system. The absorbance was measured at 540 nm using a microplate reader (Molecular Devices). Media and LPS (1 ng/mL) were used as negative and positive controls, respectively. Sodium nitrate was used as the standard curve and NO concentration was calculated using the formula bellow:

(1) $$\mathrm{NO}\;\mathrm{content}\;\left(\mu M\right)=\frac{\mathrm{Absorbance}\;\mathrm{of}\;\mathrm{sample}\;-\;0.0059}{0.0499}$$

#### 2.3.5. Cytokine Concentrations 

RAW 264.7 cells were treated with OS and incubated for 48 h. The supernatant was collected by centrifugation (15,000 rpm, 10 min, 4 °C). The immunomodulatory effects of OS in RAW 264.7 cells were evaluated by ELISA. The concentrations of cytokines (IL-1β, IFN-γ, and TNF-α) in the cell supernatant were measured by IL-1β (ab197742), IFN-γ (ab282874), and TNF-α (ab285327) according to each ELISA kit manual, respectively. An amount of 50 μL of supernatant and 50 μL of cytokine antibody cocktail were added into a 96 well plate coated with antibody and were incubated at RT for 1 h. The plate was washed three times and 100 μL of TMB solution was added to each well. It reacted for 10 min and the reaction was stopped with 100 μL of a stop solution. The absorbance was measured at 450 nm using a microplate reader (Molecular Devices). Each cytokine concentration was calculated by a standard curve.

#### 2.3.6. Cytokine Expression 

The immunomodulatory effects of OS in RAW 264.7 cells were evaluated by real time-polymerase chain reaction (RT-PCR). Total RNA was extracted from the cells using the PURY RNA Plus kit. cDNA was obtained from total RNA using the amfiRivert cDNA Synthesis Platinum Master Mix. Amplification was performed according to the conditions using the Bio-Rad CFX-96 real-time system. The primer information used for qPCR are as follows: GAPDH (QT01658692), IL-1β (QT01048355), IL-6 (QT00098875), IFN-γ (QT01038821), and TNF-α (QT00104006). GAPDH was used as a house keeping gene for normalization.

### 2.4. Animal Experiment

#### 2.4.1. Experimental Design

**Figure 1:**
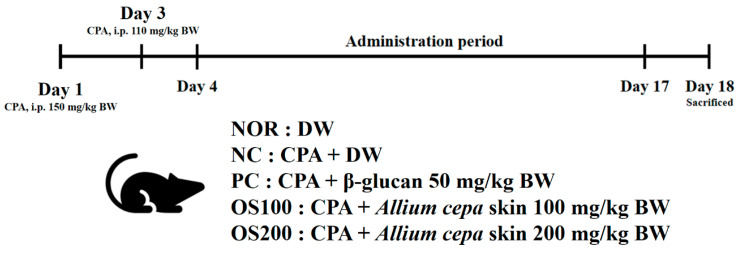
The experimental procedure.

Fifty specific pathogen free (SPF) C57BL/6 male mice (6 weeks old) were obtained from Central Lab Animal Inc. (Seoul, Korea). The mice were kept under controlled environment with temperature 23 ± 2 °C, humidity 50 ± 10%, and 12/12 h dark/light cycle. They fed normal mouse chow and water ad libitum. After 1 week of acclimatization, mice were divided into 5 groups: normal control (NOR), CPA only as a negative control (NC), CPA + β-glucan 50 mg/kg BW as a positive control (PC), CPA + OS 100 mg/kg BW (OS100), and CPA + OS 200 mg/kg BW (OS200). NOR and NC groups were orally treated with distilled water (DW) instead of β-glucan or OS. Immunosuppression was induced by 2 CPA intraperitoneal injections, which were with 150 mg/kg and 110 mg/kg body weight at 3 days and 1 day before oral administration, respectively. β-glucan and OS extract dissolved in DW were orally administered every day for 14 days (Figure 1). Body and organs weights, serum immunoglobulin and cytokine concentrations, splenocyte proliferation, and NK cell activity in the blood of the mice were measured. All experimental procedures were approved by the National Institute of Agricultural Science Committee for animal experiment (Approval Number: NAS202109).

- Group 1: NOR (normal control, distilled water (DW)) (n = 10);
- Group 2: NC (negative control, CPA, DW) (n = 10);
- Group 3: PC (positive control, CPA, β-glucan 50 mg/kg BW) (n = 10);
- Group 4: OS100 (CPA, OS extract 100 mg/kg BW) (n = 10);
- Group 5: OS200 (CPA, OS extract 200 mg/kg BW) (n = 10).

#### 2.4.2. Collecting Blood and Hematological Analysis

On the last day of this experiment, the mice were euthanized under anesthesia (CO_2_) after measuring their body weight, and the blood was collected from the orbital venous plexus. Whole blood from a mouse was placed in tube with K_2_EDTA and anti-coagulated blood was processed to determine hematological parameters (RBC, HGB, HCT, MCV, MCHC, PLT, WBC, NEU, LYM, MONO, EOS, and BASO) in a XN-hematology analyzer (Synsmex, Kobe, Japan) in accordance with manufacturer’s recommendation.

#### 2.4.3. Serum IgG, Cytokines, and NK Cell Activity

The blood sample was centrifuged (2000 rpm, 4 °C, 10 min) and the separated serum was used for the analysis of immune-related biomarkers. Immunoglobulin G (IgG) concentration in serum of the mouse was analyzed using mouse IgG ELISA kit (ab157719). The concentrations of serum cytokines (IL-1β, IFN-γ, and TNF-α) were analyzed using ELISA kits (IL-1β (ab197742), IFN-γ (ab282874), and TNF-α (ab285327)) according to the ELISA kit manual, respectively. Fifty microliters of serum and 50 μL of IgG or cytokine antibody cocktail were added into a 96 well plate coated with antibody and were incubated at RT for 2 h (IgG) and 1 h (cytokines), respectively. The plate was washed three times and 100 μL of TMB solution was added to each well. It reacted for 10 min, and the reaction was stopped with 100 μL of a stop solution. The absorbance was measured at 450 nm using a microplate reader (Molecular Devices). IgG and cytokine concentrations in serum were calculated by a standard curve, respectively. 

NK cell activity was evaluated using a murine NK cells activity ELISA test kit by measuring the level of IFN-γ secreted by the NK cell. Whole blood was mixed with heparin as an anticoagulant in test tube, 100 μL of the blood was transferred into a 96 well plate with 30 μL activator and incubated at 37 °C for 24 h. The induced plasma was obtained after centrifuge (2000 rpm, 15 min), and the level of IFN-γ was determined according to ELISA kit manual. Fifty microliters of plasma and 100 μL of antibody-enzyme conjugate were added into a 96 well plate coated with antibody and were incubated at RT for 1 h. The plate was washed four times and 100 μL of TMB solution was added to each well. It reacted for 30 min and the reaction was stopped with 100 μL of a stop solution. The absorbance was measured at 450 nm using a microplate reader (Molecular Devices). IFN-γ concentration was calculated by a standard curve.

#### 2.4.4. T-Cell and B-Cell Proliferations in Splenocytes

The spleen was washed by HBSS, put on and passed through 40 μm nylon cell strainer in 10 mL HBSS. The homogenized spleen cells were put on histopaque and centrifuged (2000 rpm, 10 min, 4 °C) to separate lymphocytes. Splenocytes were seeded at a concentration of 2 × 10^5^ cells/mL, then added with 2 μg/mL of Con A as T cell mitogen and 1 μg/mL of LPS as B cell mitogen in a 96 well plate, respectively. After incubation for 45 h, 10 μL of MTS solution was added in each well and incubated at 37 °C with 5% CO_2_ for 2 h. The absorbance was measured at 490 nm using a microplate reader (Molecular Devices).

### 2.5. Statistical Analysis 

All the samples were carried out in triplicate and analyzed using one-way analysis of variance followed by Duncan’s multiple range test (SPSS ver. 24, IBM Co., Armonk, NY, USA). Data was expressed as mean ± SEM, and values were considered as statistically significant at *p* < 0.05.

## 3. Results and Discussion

### 3.1. Functional Compounds Concentration and Antioxidant Activities of OS Extract

**Figure 2:**
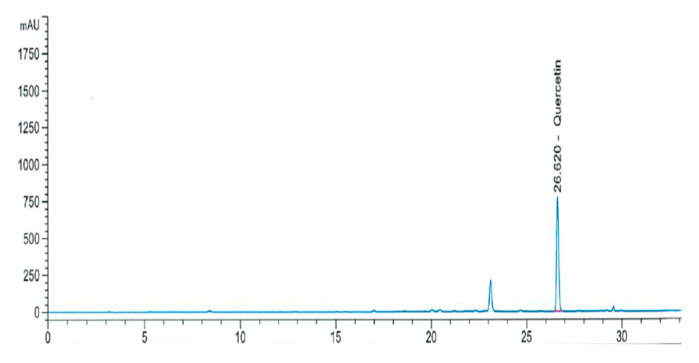
Chromatogram of quercetin in OS extract analyzed by LC.

Figure 2 shows the quercetin content in OS extract. In calibration curve, retention time was 26.627 min and correlation was 0.999. Formula was ‘Area (mAU × s) = 44.79 × amount (ug/mL) + 82.36′. For OS extract, area and amount were 5856.0 mAU × s and 128.89 μg/mL, and the quercetin concentration of OS extract was 37.9 ± 0.6 mg/g (Table 2). Quercetin is the main functional component in onions and has diverse biological activities including anti-oxidant, anti-diabetic, and anti-obesity [21]. Quercetin content of onion skin extract produced by ethanol, hot water, and subcritical water extractions at 110 °C and 165 °C were 62.4 ± 1.2, 25.8 ± 1.8, 44.4 ± 1.0, and 12.3 ± 2.8 mg/g dry weight, respectively [22]. Our result showed that the quercetin content (37.9 mg/g) in OS extract used in this study was 46.9% higher than that (25.8 mg/g) of hot water extract from onion skin in the previous study. 

Total phenolic content (TPC) of OS extract was 65.6 ± 0.1 mg GAE/g as shown in Table 2. In a previous study, TPCs of OS extracts by ethanol, hot water, and subcritical water ranged from 56.7 mg GAE/g to 372.5 mg GAE/g [22]. The values of Indian OS methanol extracts were between 14.55 mg GAE/g and 289.04 mg GAE/g [13]. TPCs of red, white, and yellow onion extracts produced by methanol were 132 mg GAE/mL, 112 mg GAE/mL, and 120 mg GAE/mL, respectively [23]. OS was rich in quercetin, phenolics, and flavonoids, and these compounds have been reported with high antioxidant capacity [24]. Our result showed that the TPC in OS extract was lower than that in ethanol extract from OS [22]. The amounts of antioxidant compounds were dependent on extracting solvents, and polar solvents are generally used to extract polyphenols from a plant matrix [25].

DPPH radical is reduced to DPPH-H by the reaction with an antioxidant [26], and the DPPH radical scavenging activity of OS ranged from 14.78% to 68.07% at 62.5–2000 μg/mL (Table 3). The IC_50_ value of OS was 945.7 μg/mL. OS extract in DPPH radical scavenging activity showed significant increase in a dose dependent manner. Previous research reported that DPPH radical scavenging activity of OS ethanolic extract was 28.39% at 1 mg/mL [27], while it was 46.89% at the same concentration in our study. OS extracts produced by ethanol, hot water, and subcritical water showed the different DPPH radical scavenging activities with 72.25%, 49.68%, and 64.72%, respectively [22]. DPPH radical scavenging activity of ethanolic extract from whole onion was 18.47% [28] and its IC_50_ value was 43.24 μg/mL [29]. Thus, the antioxidant effects of OS were influenced by extraction solvents. Previous study showed that that the solvents contributed to antioxidant activity and solvents with high polarity expressed higher antioxidant activity [30]. 

An antioxidant reduced ABTS^·+^ to the substrate ABTS [31]. This assay is commonly used for analysis of antioxidant compound activity [32]. ABTS radical scavenging activity of OS extract ranged from 76.85% to 99.68% (Table 3). The IC_50_ value of OS was 26.5 μg/mL. ABTS radical scavenging activity of OS increased in a dose-dependent manner. ABTS radical scavenging activity of OS ethanolic extraction was 33.29% in 1 mg/mL [27]. The high contents of total polyphenol in Indian OS cultivars showed the high antioxidant activities in DPPH and ABTS assays [13]. OS extract presented higher ABTS radical scavenging activity than DPPH radical scavenging activity, and it may be explained that ABTS radical scavenging activity was affected by both hydrophilic and lipophilic compounds [30]. It has been reported that pigments in foods made the difference between DPPH and ABTS assays [33]. DPPH radical scavenging activity was evaluated in organic media, while ABTS activity was analyzed in aqueous and ethanolic media. ABTS reacted in wide pH range and was not affected by ionic strength [34]. For these reasons, OS may show higher ABTS radical scavenging activity than DPPH radical scavenging activity. TPC had the relationship with DPPH and ABTS radical scavenging activities, and the greater proportion of polyphenols was expected to be correlated with the high antioxidant activity [35]. 

XO is an enzyme involved in purine metabolism, converts hypoxanthine to xanthine and xanthine to uric acid using oxygen, and causes kidney disease and gout [36]. Inhibition of XO reduces the production of oxygen radicals [37]. OS extract significantly improved XO inhibitory activity in a dose dependent manner and IC_50_ value of OS was 10.7 μg/mL (Table 4). The XO inhibitory rate of onion was over 90% and IC_50_ value was 17.36 μg/mL [31]. A previous study demonstrated that the concentration of flavonols in OS was positively correlated with the decrease in XO activity, and XO inhibitory activities (IC_50_ values) of flavonol fractions from OS were between 0.67 μg/mL and 0.95 μg/mL [38]. IC_50_ values of onion solid waste ranged from 15.2 μg/mL to 35.2 μg/mL [39]. OS extract showed higher XO inhibitory activity with lower IC_50_ value (10.7 μg/mL) compared with those of IC_50_ (15.2–35.2 μg/mL) in the onion solid waste including apical trimmings [39]. Phenolic compounds exerted the inhibitory activity against XO [40]. In this study, antioxidant capacities, including DPPH and ABTS, radical scavenging activities and XO inhibitory activity might be affected by high amounts of quercetin and TPC in OS extract.

### 3.2. Effects of OS Extracts on Antioxidant Enzymes in RAW 264.7 Cells

**Figure 3:**
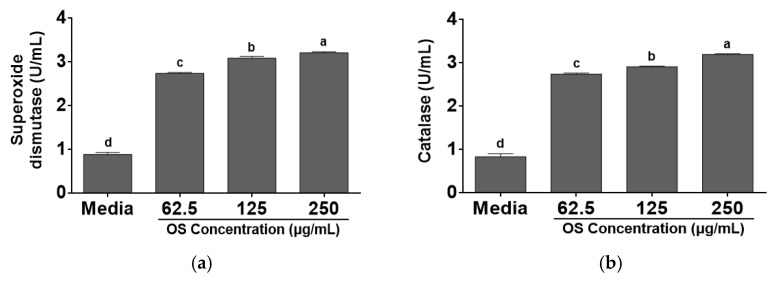
Antioxidant enzymes activities of *A. cepa* skin (OS) extracts in RAW 264.7 cells. (**a**) Superoxide dismutase activity (SOD); (**b**) catalase activity (CAT). Data was expressed as the mean ± SEM. ^a–d^ Different letters on bars are significantly different among SOD or CAT values at different concentrations (*p* < 0.05).

SOD and CAT are the main antioxidant enzymes. SOD catalyzes a dismutation of the superoxide anion to hydrogen peroxide, which is reduced to water and molecular oxygen by CAT [41]. OS increased SOD and CAT activities in RAW 264.7 cells compared with media. SOD and CAT activities ranged from 0.88 ± 0.10 U/mL to 3.21 ± 0.06 U/mL, and from 0.83 ± 0.17 U/mL to 3.20 ± 0.04 U/mL, respectively, at 62.5–250 μg/mL of OS extract (Figure 3). Previous study evaluated antioxidant enzymes in Wistar rats and identified that treatment with *A. cepa* significantly increased the levels of SOD and CAT [42]. Diabetic rats fed the bread supplemented with OS extract and onion powder showed increased activities in SOD and CAT in kidney and liver compared with the control group [43]. Ageing rats treated with OS ethanolic extract showed higher SOD and CAT activities in plasma, liver, and brain than negative control which was not treated with OS ethanolic extract [44]. There was a significant increase in SOD and CAT activities in OS treated cells compared with the media control, and it was a similar result with previous studies. Therefore, OS can eliminate oxygen radicals in the body and be a useful source as antioxidant supplement.

### 3.3. Effects of OS Extract on Cell Viability and NO Production by RAW 264.7 Cells

**Figure 4:**
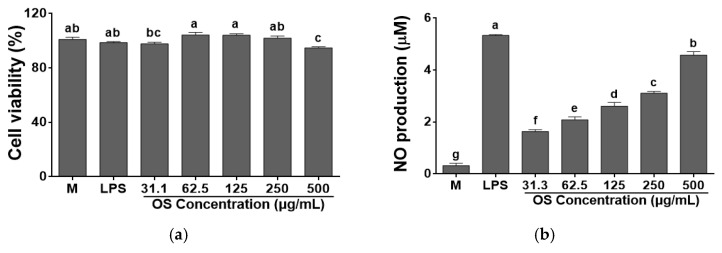
Effects of *A. cepa* skin (OS) extracts on (**a**) cell viability and **(b)** the nitric oxide (NO) production by RAW 264.7 cells. Data was expressed as the mean ± SEM. ^a–g^ Different letters on bars are significantly different at *p* < 0.05.

To assess whether the OS affects cell viability in various concentrations (62.5–500 μg/mL), we conducted MTS assay. OS was not cytotoxic on RAW 264.7 cells at less than 500 μg/mL compared to media (Figure 4a). Hot water extract of onion skin also did not show any cytotoxicity in RAW 264.7 cells, which was evaluated by MTT assay [14]. 

NO is the inflammatory molecule which can kill pathogens and tumor cells. NO is released when the macrophage was activated [45]. The immune stimulating effect of OS was performed by measuring the content of NO in RAW 264.7 cells. NO content ranged from 1.6 μM to 4.6 μM at 31.3–500 μg/mL (Figure 4b), respectively. Treatment with hot water extract from OS significantly improved NO content in RAW264.7 cells compared with media. Previous study reported that the NO production of onion and methanol extract from onion was 1.09 μM and 1.41 μM at 1000 μg/mL, respectively [46]. *A. hookeri* stimulated the production of NO in RAW 264.7 cells [18,47]. NO regulates the adaptive immune response and depresses or stimulates the pro-inflammatory cytokine expression [48,49]. Thus, these results indicated that OS could enhance the NO production in RAW 264.7 macrophage cells and OS may be more efficient in NO generating than onion.

### 3.4. Effects of OS Extracts on Cytokine Productions by RAW 264.7 Cells

**Figure 5:**
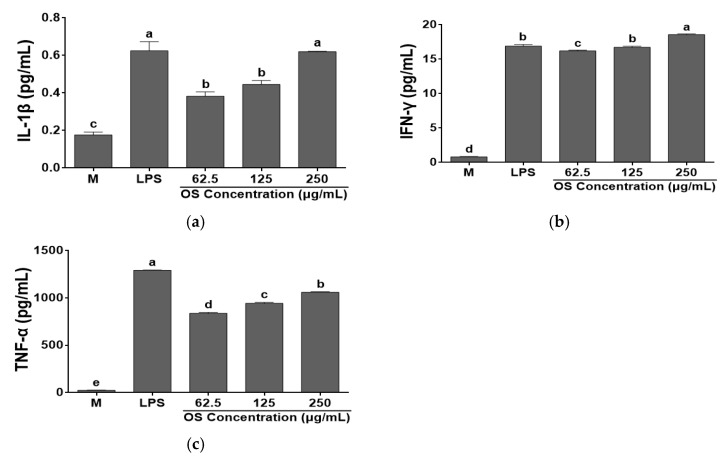
Effects of *A. cepa* skin (OS) extracts on the productions of (**a**) IL-1β, (**b**) IFN-γ, and (**c**) TNF-α by RAW 264.7 cells. Data was expressed as the mean ± SEM. ^a–e^ Different letters are significantly different at *p* < 0.05.

**Figure 6:**
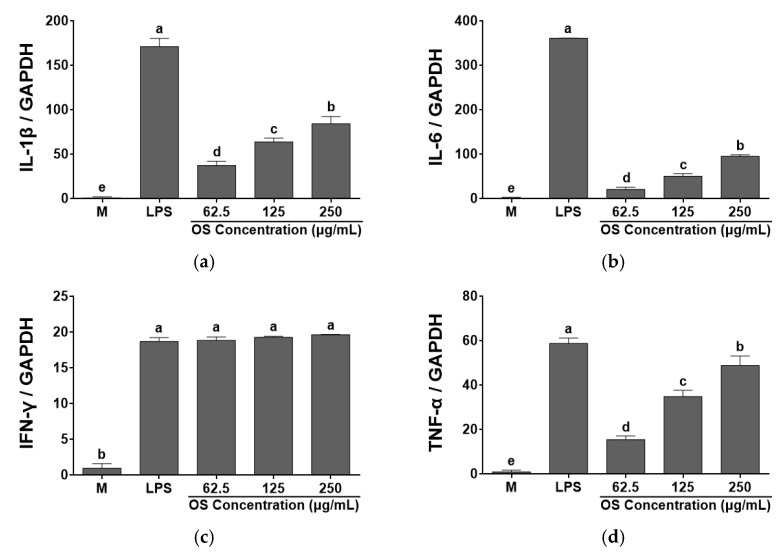
Effects of *A. cepa* skin (OS) extracts on the expressions of (**a**) IL-1 β, (**b**) IL-6, (**c**) IFN- γ, and (**d**) TNF-α by RAW 264.7 cells. Data was expressed as the mean ± SEM. ^a–e^ Different letters are significantly different at *p* < 0.05.

This study evaluated the relationship between OS and the production of cytokines (Figure 5 and Figure 6). Cytokine levels (IL-1β, IFN-γ, IL-6, and TNF-α), which are typical immune related indicators, were measured by ELISA. IL-1β is involved in T cell activation, B cell maturation and NK cell activation [50]. IL-6 is a B cell growth factor that induces hepatocytes to synthesize some plasma proteins, such as fibrinogen. Additionally, it induces B cell differentiation and promotes the synthesis of immunoglobulin and a synergistic action in cooperation with other cytokines [51]. IL-6 was not detected in the cell supernatant by ELISA assay. However, OS significantly increased IL-1β, IFN-γ, and TNF-α levels produced by RAW 264.7 cells in a dose dependent manner, compared to the media. IFN-γ is one of the major products of T-helper type 1 cells (Th1). It enhances the secretion of NK cells against pathogens and viruses [52]. TNF-α is a cytokine that destroys tumor cells and it interacts with T lymphocytes to regulate the activity and growth of T lymphocytes, and may exert a direct anticancer effect by inducing cytolysis of cancer cells [53].

OS extract significantly increased gene expressions of IL-1β, IL-6, IFN-γ, and TNF-α in a dose dependent manner (Figure 6). IL-6 expression was found by RT-PCR assay though it was not detected in the cell supernatant by ELISA assay. Based on the results, OS extract activated RAW 264.7 cells and dose-dependently improved expressions of cytokine IL-1β, IL-6, IFN-γ, and TNF-α. The data indicated that OS played an important role in cytokine secretion. In the previous studies, *A. fistulosum* and *A. cepa* agglutinin improved TNF-α and IFN-γ in RAW 264.7 cells [54], *A. cepa* increased the level of IL-6, IFN-γ, and TNF-α, and its stimulatory effects on Th1 activity were indicated [32]. However, *A. cepa* ethanol extract inhibited IL-6 secretion and whole onion with leaves suppressed the concentration of cytokines (IL-1β, IL-6, and TNF-α) in RAW 264.7 cells treated with LPS [28,53]. Thus, *A. cepa* may control immune system by stimulating or depressing cytokine expression according to the host or LPS-treated conditions. OS extract can keep Th1 and Th2 balance by modulating pro- and anti-inflammatory cytokines. In this study, OS extract significantly increased the expressions of cytokines in a dose dependent manner by activating RAW 264.7 cells, and the results were similar to the previous studies. Therefore, the results indicate that OS could stimulate the immunity response of RAW 264.7 cells, and it could be used as a prospective functional material.

### 3.5. Effects of OS Extracts on Body Weight Gain and Hematological Factors of the Immunosuppressed Mice 

Body weight gain (BWG, g) significantly decreased in the NC group compared to the NOR control group. However, BWG was higher in PC, OS100, and OS200 groups, and BWG in OS200 group was recovered to that of the NOR group (Table 5). The hematological data was also shown in Table 5. In this study, white blood cells (WBC) numbers of NC group were significantly lower than those of the other groups. The counts of WBC and eosinophil significantly increased after OS treatment compared to the NC group. The number of WBC in OS200 group was recovered to that of the NOR group (*p* > 0.05) and it was higher than that of PC group (*p* < 0.05). CPA induced hemorrhagic cystitis and hematopoietic depression, including leukocytes and platelets [55]. *Phellinus baumii* showed immune-protective effect in CPA-induced mice [55]. WBC, lymphocytes, and platelet counts significantly increased due to the administration of *A. cepa* agglutinin in immunosuppressed the CFT Wistar rats treated with CPA [56]. In the present study, the results showed that OS could improve decreased body weight and the hematological factors of the mice with CPA-induced immunosuppression and effectively encourage immune-enhancing in the model.

### 3.6. Effects of OS Extracts on Serum Immunoglobulin and NK Cell Activity in Immunosuppressed Mice

**Figure 7:**
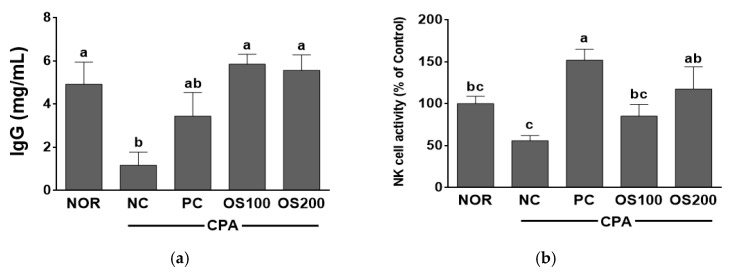
Effects of *A. cepa* skin (OS) extract on the serum (**a**) IgG level and (**b**) NK cell activity in C57BL/6 mice immunosuppressed by cyclophosphamide (CPA). NOR, normal control group; NC, negative control group; PC, positive control group; OS100, OS extract 100 mg/kg BW; OS200, OS extract 200 mg/kg BW. Data was expressed as the mean ± SEM. ^a–c^ Different letters are significantly different at *p* < 0.05.

The serum IgG level was shown in Figure 7a. Serum IgG levels in OS100 and OS200 groups were 5.85 ± 0.42 mg/mL and 5.56 ± 0.55 mg/mL, respectively, they are higher than that of PC (3.44 ± 0.78 mg/mL). The IgG level in OS groups treated at 100 mg/kg and 200 mg/kg BW increased compared to the NC group and was similar to the level of NOR which was not treated with CPA. Immunoglobulin is a protein that reacts specifically with antigens that stimulate their production and inactivates pathogens to get rid of them. IgG is a main immunoglobulin that has a role of continuous defense against infection with phagocytosis of macrophage. Additionally, it recognizes and eliminates pathogens and toxic antigens [57]. The treatment with tremella polysaccharides recovered the IgG level in CPA-induced immunosuppressed model [58]. The extract of polysaccharide from *Strongylocentrotus nudus* eggs increased IgA, IgG, and IgM [59]. In the present study, the administration of OS stimulated the production of IgG in the immune-depressed mice and it might enhance immune system. 

As shown in Figure 7b, NK cell activities of NC, PC, OS100, and OS200 groups were 56.03 ± 6.00%, 151.88 ± 13.04%, 85.22 ± 13.98%, and 117.46 ± 26.41%, respectively. All groups showed higher values than NC group and specially increased in OS groups in a dose dependent manner. PC and OS200 showed the highest values and they were higher than or similar to that of NOR group (100.00 ± 8.89%). The results demonstrated that OS stimulates NK cell activity, induces secretion of IFN-γ, and controls balancing cytokines secreted by Th1 and Th2. NK cells are crucial cells of the innate immune system, activated NK cells destroy target cells by receptor-ligand interaction and act an immune response to pathogens entering the body [60]. Treatment with water and ethanol extracts from *A. hookeri* increased the NK cell activity in type 2 C57BL/J-*db/db* mice [61]. An oral administration of *A. fistulosum* micus to the older mice activated splenic NK cells [54]. *P. baumii* extract had the potential to prevent CPA-induced immunosuppression in splenic and peritoneal NK cell activities [55]. In this study, OS extract enhanced the production of IFN-γ by stimulating NK cell activity. Therefore, OS may effectively contribute to the immune system by recovering NK cell activity in the mice with CPA-induced immune depression.

### 3.7. Effects of OS Extracts on Serum Cytokine Concentration in Immunosuppressed Mice

**Figure 8:**
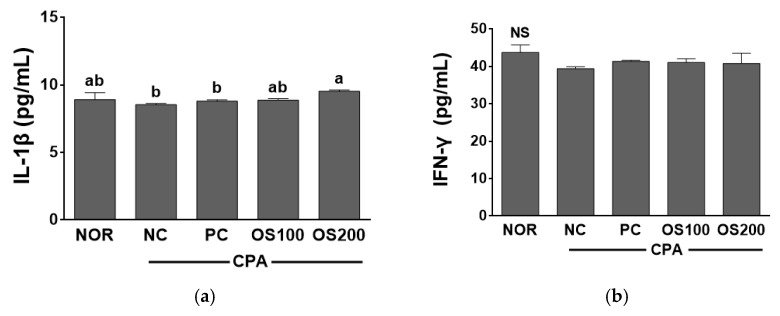
Effects of *A. cepa* skin (OS) extract on the serum (**a**) IL-1β and (**b**) IFN-γ levels in C57BL/6 mice immunosuppressed by cyclophosphamide (CPA). NOR, normal control group; NC, negative control group; PC, positive control group; OS100, OS extract 100 mg/kg BW; OS200, OS extract 200 mg/kg BW. Data was expressed as the mean ± SEM. ^a,b^ Different letters are significantly different at *p* < 0.05. ^NS^ Not significantly different.

Serum Th1 cytokine levels (IL-1β and IFN-γ) related with cell mediated immunity were evaluated and were shown in Figure 8. CPA is an immunosuppressant and induces to decrease the count of T cells and production of cytokines [59]. The levels of IL-1β and IFN- γ were lower in NC compared to the NOR group due to CPA administration (*p* > 0.05). IL-1β levels of NOR, NC, PC, OS100, and OS200 groups were 8.92 ± 0.50 pg/mL, 8.53 ± 0.10 pg/mL, 8.79 ± 0.11 pg/mL, 8.87 ± 0.11 pg/mL, and 9.52 ± 0.11 pg/mL, respectively. The serum IL-1β levels in OS100 and OS200 groups were higher than that of NC group. OS200 group showed a significantly higher IL-1β value than that of NC group. IFN-γ values of NOR, NC, PC, OS100, and OS200 were 43.7 ± 2.0 pg/mL, 39.4 ± 0.6 pg/mL, 40.1 ± 1.1 pg/mL, 41.0 ± 1.0 pg/mL, and 41.9 ± 3.5 pg/mL, respectively. There was no significant difference in IFN-γ level among all groups though NC group showed relatively lower value than NOR group. OS100 and OS200 showed higher IFN-γ level than PC group treated with β-glucan (*p* > 0.05). *P. baumii* improved the levels of IL-1β and IFN-γ in CPA-induced immunosuppressed mice [55]. Recent studies showed that the supplementation of onion lectin in rats improves TNF-α and IL-10 values [53]. The production of IFN-γ was enhanced in the murine thymocytes treated with onion lectin [62]. However, the biological activity of *A. cepa* extract in the serum of Sprague Dawley rats showed that pro- and anti-inflammatory cytokines were not affected by *A. cepa* [63]. In previous studies, OS effectively attributed to the production of cytokines than *A. cepa* flesh. It may be due to its higher concentration of quercetin and TPC concentrations in the onion skin. Therefore, OS may enhance immunity by producing serum IL-1β and IgG levels and by improving NK cell activities in the immunosuppressed mice.

### 3.8. Effects of OS Extracts on the Proliferation of Mice Splenocytes

**Figure 9:**
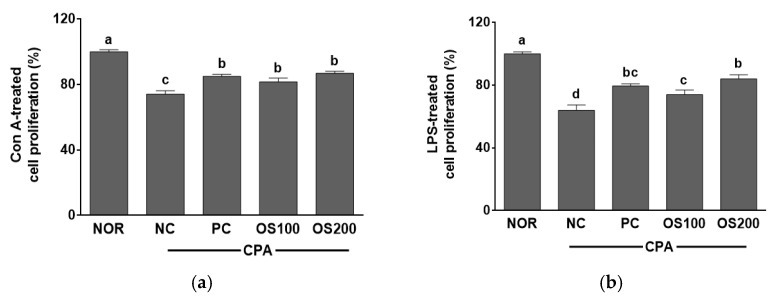
Effects of *A. cepa* skin (OS) extracts on the proliferation of splenocytes treated with (**a**) Con A and (**b**) LPS in the C57BL/6 mice immunosuppressed by cyclophosphamide (CPA). NOR, normal control group; NC, negative control group; PC, positive control group; OS100, OS extract 100 mg/kg BW; OS200, OS extract 200 mg/kg BW. Data was expressed as the mean ± SEM. ^a–d^ Different letters are significantly different at *p* < 0.05.

This study evaluated the proliferation of splenocytes to confirm the effect of OS in the growth of immune cells (Figure 9). OS showed higher splenocytes proliferation when cultured with Con A than the NC group. OS100 and OS200 groups also did similar levels compared with the PC group after incubation with Con A as T cell mitogen. In the splenocytes treated with LPS as B cell mitogen, the proliferation in NC group was the lowest among five groups. It was higher in OS100 group than NC group and in OS200 than PC group. The cell proliferation treated with Con A or LPS had an analogic tendency in this study. The spleen, a lymphoid organ, constitutes the main filter of the body of blood-borne antigens, and the site of differentiation and homing of inflammatory macrophages, granulocytes, and NK cells [64]. In the previous study, the extract of *A. hookeri* leaves and roots improved the proliferation of splenocytes [18]. The polysaccharide of *Cyclocarya paliurus* recovered the proliferation of splenocytes in CPA-induced immunosuppression mice model in the presence of Con A and LPS [57]. Additionally, the splenocytes proliferation of rat that administrated with onion lectin significantly increased compared to the untreated control [56]. From the results, it is suggested that OS may induce T and B cell proliferation in splenocytes and have the potential to enhance the immune system by immune-stimulating effects in T and B lymphocytes.

## 4. Conclusions

This study demonstrated that OS extract is a good material with antioxidant and immune enhancing effects. OS showed strong antioxidant effects and effectively stimulated the immune system by increasing the production of NO and cytokines (IL-1β, IL-6, IFN-γ, and TNF-α) in RAW 264.7 cells. In the animal experiment using immunosuppressed mice, the count of WBC, and T-cell and B-cell proliferations were higher in OS100 and OS200 groups than in NC group. Additionally, serum IgG, NK cell activity, and IL-1β levels were higher in OS200 group compared to the NC group. Therefore, verifying functional effects of OS and developing its usage can contribute to both improving public health and protecting environment from pollution through carbon neutrality and upcycling of agro-food by-products. 

## Figures and Tables

**Table 1:** HPLC condition for quercetin analysis.

	Condition		
Instrument	Agilent 1260 Infinity Binary LC		
Column	Zorbax Eclipse Plus C18 UG 120 (4.6 × 250 mm, 5 μm)		
Mobile phase	Mobile phase A: 5% formic acid Mobile phase B: MeOH		
	Time (min)	A (%)	B (%)
	0	100	0
	25	40	60
	26	0	100
	30	40	60
	31	80	20
	37	80	20
Gradient program	Gradient elution of mobile phase A and B		
UV length	360 nm		
Injection volume	10 μL		
Column temperature	40 °C		
Flow rate Run time	0.8 mL/min 37 min		

**Table 2:** Quercetin concentration and total phenolic content of *A. cepa* skin (OS) extracts.

Sample	Quercetin (mg/g)	Total Phenolic Content (mg GAE/g)
OS extract	37.9 ± 0.6	65.6 ± 0.1

**Table 3:** DPPH and ABTS radical scavenging activities of *A. cepa* skin (OS) extracts.

Sample	Concentration (μg/mL)	DPPH Radical Scavenging Activity (%)	ABTS Radical Scavenging Activity (%)
OS extract	62.5	14.78 ± 0.44 ^f^	76.85 ± 0.23 ^e^
	125	18.67 ± 0.26 ^e^	90.78 ± 0.12 ^d^
	250	26.53 ± 0.46 ^d^	95.90 ± 0.09 ^c^
	500	36.19 ± 0.93 ^c^	98.82 ± 0.06 ^b^
	1000	46.89 ± 0.94 ^b^	99.68 ± 0.06 ^a^
	2000	68.07 ± 0.99 ^a^	99.68 ± 0.07 ^a^
	IC_50_ ^(1)^	945.7 ± 32.3	26.5 ± 0.2

Data was expressed as mean ± SEM. ^a–f^ Different letters are significantly different in each column at *p* < 0.05. ^(1)^ IC_50_: The concentration (μg/mL) required for 50% reduction in DPPH radical or ABTS radical.

**Table 4:** Xanthine oxidase inhibitory activity of *A. cepa* skin (OS) extracts.

Sample	Concentration (μg/mL)	Xanthine Oxidase Inhibitory Activity (%)
OS extract	6.3	32.20 ± 0.87 ^d^
	12.5	53.18 ± 0.34 ^c^
	25	75.70 ± 0.29 ^b^
	50	89.74 ± 0.70 ^a^
	100	90.66 ± 0.76 ^a^
	IC_50_ ^(1)^	10.7 ± 0.2

Data was expressed as mean ± SEM. ^a–d^ Different letters are significantly different in each column at *p* < 0.05. ^(1)^ IC_50_: The concentration (μg/mL) required for 50% reduction in xanthine oxidase.

**Table 5:** Effects of *A. cepa* skin (OS) extracts on the body weight gain and hematological factors in C57BL/6 mice immunosuppressed by cyclophosphamide.

Group		NOR ^(1)^	NC	PC	OS100	OS200
Body weight gain (g/mice) Red blood cell (RBC, ×10^6^ cells/μL)		2.74 ± 0.39 ^a^ 10.2 ± 0.2 ^ab^	0.98 ± 0.26 ^b^ 9.98 ± 0.10 ^ab^	1.28 ± 0.26 ^b^ 9.97 ± 0.13 ^ab^	1.30 ± 0.21 ^b^ 9.85 ± 0.16 ^b^	1.93 ± 0.21 ^ab^ 10.3 ± 0.1 ^a^
Hemoglobin (Hb, g/dL)		14.9 ± 0.3 ^NS^	14.4 ± 0.2	14.6 ± 0.2	14.3 ± 0.2	14.8 ± 0.1
RBC indexes	MCV (fL) ^(2)^	49.5 ± 0.6 ^b^	48.7 ± 0.5 ^b^	49.5 ± 0.5 ^bc^	49.6 ± 0.2 ^b^	51.6 ± 0.5 ^a^
	MCH (pg)	14.6 ± 0.0 ^ab^	14.5 ± 0.0 ^abc^	14.6 ± 0.1 ^a^	14.3 ± 0.1 ^c^	14.41 ± 0.0 ^bc^
	MCHC (g/dL)	29.8 ± 0.3 ^a^	29.6 ± 0.3 ^ab^	29.0 ± 0.3 ^abc^	28.8 ± 0.2 ^bc^	28.5 ± 0.3 ^c^
Hematocrit (%)		50.3 ± 1.1 ^b^	47.8 ± 0.6 ^c^	49.7 ± 0.4 ^bc^	49.3 ± 0.5 ^bc^	52.4 ± 0.3 ^a^
Platelet (×10 ^3^ cells/μL)		1226 ± 44 ^NS^	907.9 ± 104.8	993.8 ± 116.8	1010 ± 97	1026 ± 107
White blood cell (WBC, ×10^3^ cells/μL)		4.40 ± 0.33 ^a^	2.36 ± 0.43 ^c^	3.05 ± 0.44 ^bc^	3.29 ± 0.29 ^b^	4.37 ± 0.21 ^a^
WBC differential counting (%)	Neutrophil	7.53 ± 0.07 ^b^	19.8 ± 0.8 ^a^	15.6 ± 0.4 ^a^	15.4 ± 0.9 ^a^	18.7 ± 2.7 ^a^
	Lymphocyte	83.1 ± 0.8 ^a^	68.7 ± 2.5 ^b^	74.8 ± 2.0 ^b^	75.1 ± 1.7 ^b^	73.9 ± 2.0 ^b^
	Monocyte	3.67 ± 0.52 ^NS^	3.78 ± 0.31	3.98 ± 0.41	4.18 ± 0.63	3.88 ± 0.55
	Eosinophil	7.43 ± 0.74 ^a^	1.73 ± 0.67 ^b^	5.30 ± 0.86 ^a^	7.47 ± 1.48 ^a^	5.27 ± 1.64 ^a^
	Basophil	0.12 ± 0.05 ^NS^	0.00 ± 0.00	0.08 ± 0.05	0.12 ± 0.07	0.10 ± 0.04

^(1)^ NOR, normal control group; NC, negative control group; PC, positive control group; OS100, OS extract 100 mg/kg BW; OS200, OS extract 200 mg/kg BW. ^(2)^ MCV, mean corpuscular volume; MCH, mean corpuscular Hb; MCHC, mean corpuscular Hb concentration. Data was expressed as the mean ± SEM. ^a–c^ Different letters are significantly different at *p* < 0.05. ^NS^ Not significantly different.

## Data Availability

Data is contained within the article.
